# Immunometabolic synaptopathy: linking metabolic syndrome to epileptogenesis through trained immunity and parvalbumin interneuron vulnerability

**DOI:** 10.3389/fncel.2026.1979155

**Published:** 2026-10-09

**Authors:** Baihui Zhang, Wenjin Wang, Tao Li, Hua Huang

**Affiliations:** 1 School of Medical and Life Science, Chengdu University of Traditional Chinese Medicine, Chengdu, China; 2 Department of Neurology, Dazhou Central Hospital, Dazhou, China

**Keywords:** CNS border immunity, epileptogenesis, meningeal lymphatic system, metabolic syndrome, microglial priming, parvalbumin interneurons, perineuronal nets, trained immunity

## Abstract

**Background:**

Metabolic syndrome (MetS) and epilepsy are clinically associated, but how chronic peripheral metabolic stress might influence seizure susceptibility remains unclear. The potential links among CNS-interface dysfunction, glial responses, and inhibitory-circuit vulnerability require further clarification.

**Proposed framework:**

We propose immunometabolic synaptopathy as a hypothesis-driven framework linking chronic metabolic dysfunction to impaired inhibitory-circuit resilience. MetS-associated metabolic and inflammatory signals may alter blood–brain barrier function, CNS-border immune signaling, and glial homeostasis. Evidence from peripheral myeloid training and CNS immune-memory models supports the plausibility of trained-immunity-like microglial priming, but its occurrence and persistence in MetS-associated epileptogenesis remain unestablished.

**Circuit-level convergence:**

We identify the parvalbumin-positive interneuron–perineuronal net (PV-PNN) unit as a candidate convergence point because of its energetic demands and dependence on ionic and extracellular-matrix homeostasis. Altered metabolic and trophic support, oxidative stress, impaired K^+^ and glutamate handling, and PNN remodeling may jointly compromise PV-mediated inhibition. Whether these processes preferentially affect PV-PNN units or precede spontaneous seizures requires direct testing.

**Therapeutic implications:**

Five candidate nodes—NLRP3, mTOR, HIF-1α, MMP-9, and Kir4.1—provide opportunities for mechanistic testing. Their therapeutic relevance to MetS-associated epilepsy remains uncertain, and available compounds differ in selectivity, CNS exposure, and safety. The framework motivates experimental evaluation of intervention timing and target dependence rather than specific clinical treatment recommendations.

**Conclusion:**

Immunometabolic synaptopathy integrates cross-context evidence into a testable hypothesis of metabolically associated seizure susceptibility. The complete pathway to spontaneous epilepsy has not been demonstrated end-to-end and requires longitudinal, cell- and pathway-specific validation.

## 1 Introduction

Epilepsy and metabolic syndrome (MetS) frequently coexist and show bidirectional clinical associations. Large population-based cohort studies have demonstrated that type 2 diabetes, obesity, and related metabolic abnormalities are associated with an increased risk of new-onset epilepsy (Lu et al., 2018; Dafoulas et al., 2017; Johnson et al., 2018), whereas individuals with epilepsy have a higher prevalence of metabolic comorbidities than the general population (Gaitatzis et al., 2012; Janousek et al., 2013; Ladino et al., 2014). Previous reviews have summarized several mechanisms that may contribute to this relationship, including systemic inflammation, blood–brain barrier (BBB) dysfunction, gut dysbiosis, insulin resistance, and metabolic effects related to antiseizure medications (Devinsky et al., 2018; Pandey and Raman, 2021). However, these mechanisms are commonly presented as parallel or partially overlapping mechanisms, and it remains unclear how chronic peripheral metabolic stress might be translated into a persistent and circuit-selective state of neuronal hyperexcitability.

Importantly, direct causal evidence linking MetS to epilepsy through a continuous pathway from peripheral metabolic dysfunction to selective neuronal impairment remains limited. Evidence supporting individual components of this putative pathway derives from epidemiological studies, experimental models of metabolic disease, neuroinflammation, and epilepsy, but relatively few studies have interrogated the entire sequence within a single experimental system. Therefore, the proposed relationships should not be interpreted as an established linear causal cascade. Throughout this review, we distinguish well-supported biological mechanisms from cross-context mechanistic inference and explicitly hypothesis-generating links that require further experimental validation.

Here, we propose immunometabolic synaptopathy as a hypothesis-driven and experimentally testable framework for integrating these observations. In this model, chronic metabolic disturbances—including hyperglycemia, dyslipidemia, obesity-associated inflammation, gut-derived microbial signals, and altered myelopoiesis—recurrently challenge CNS immune and metabolic interfaces, including the BBB, the meningeal lymphatic-glymphatic-skull marrow axis, and gut-brain immune/metabolic signaling pathways. These abnormalities may increase or prolong CNS exposure to metabolic and inflammatory mediators and promote trained-immunity-like priming of myeloid cells and microglia together with astrocytic homeostatic failure. These glial alterations may subsequently converge on the parvalbumin-positive interneuron-perineuronal net (PV-PNN) unit through bioenergetic, oxidative, ionic, trophic, and extracellular-matrix disturbances, thereby weakening perisomatic inhibition and promoting excitation-inhibition (E/I) imbalance and network hyperexcitability (Figure 1).

**Figure 1:**
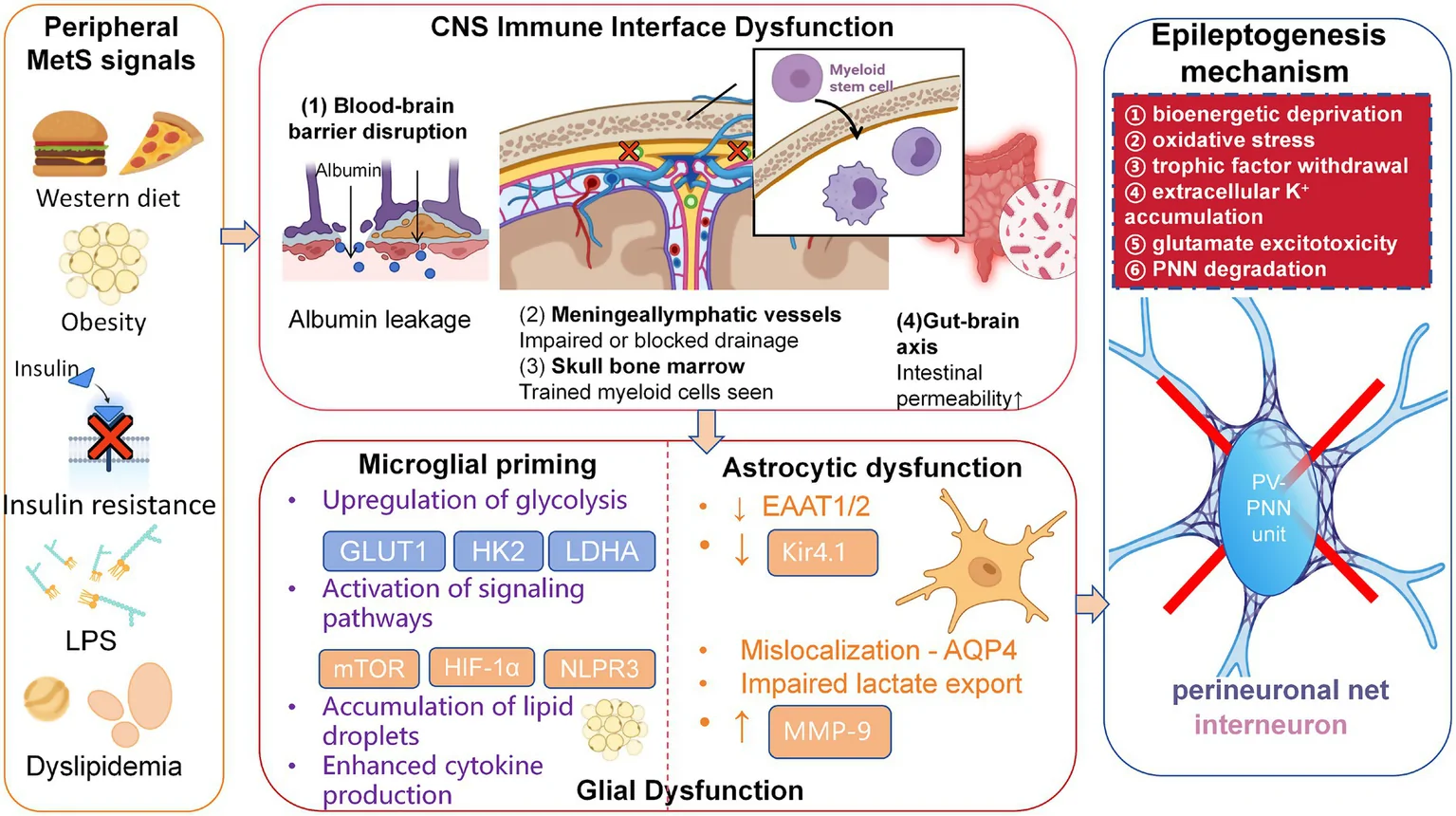
The immunometabolic synaptopathy framework in metabolic syndrome-associated epileptogenesis.

Rather than proposing a universal mechanism applicable to all forms of epilepsy, this framework is intended to describe one biologically plausible pathway through which chronic metabolic dysfunction may increase epileptogenic susceptibility in a subset of vulnerable individuals. Its principal value lies in integrating previously fragmented observations into a structured sequence of mechanistic nodes that can be experimentally tested, refined, or falsified, while also providing a basis for future phenotype-specific and translational studies.

The schematic integrates proposed interactions among peripheral metabolic disturbances, CNS interfaces, glial responses, and inhibitory-circuit vulnerability. Peripheral signals associated with metabolic syndrome (MetS), including dyslipidemia, insulin resistance, and gut-derived inflammatory signals, may alter blood–brain barrier function, CNS-border immune signaling, and fluid clearance. Skull marrow–meningeal myeloid trafficking represents a potential route of border immune communication; whether MetS induces trained phenotypes in cells using this route remains unknown. Candidate glial responses include trained-immunity-like microglial priming and altered astrocytic glutamate uptake, K^+^ buffering, metabolic support, and extracellular-matrix regulation. Six potentially interacting stress pathways—altered energy-substrate availability, oxidative stress, altered trophic signaling, extracellular K^+^ accumulation, excess glutamatergic signaling, and perineuronal net (PNN) remodeling—may compromise the resilience of the parvalbumin-positive interneuron–PNN (PV-PNN) unit and increase seizure susceptibility. NLRP3, mTOR, HIF-1α, MMP-9, and Kir4.1 are highlighted as candidate nodes for experimental interrogation. Connections represent proposed relationships rather than a demonstrated causal sequence or quantified contributions; the complete pathway to spontaneous epilepsy remains unvalidated.

## 2 CNS immune interfaces in MetS-associated epileptogenesis

Metabolic syndrome (MetS) is characterized by chronic low-grade systemic inflammation (“meta-inflammation”) accompanied by hyperglycemia, insulin resistance, dyslipidemia, altered adipokine signaling, circulating inflammatory mediators, and gut-derived microbial products. Rather than acting directly on neuronal circuits as a single unified insult, these peripheral abnormalities must interact with anatomical and functional interfaces that regulate communication between the circulation, cerebrospinal fluid (CSF), meninges, and brain parenchyma.

In the framework proposed here, three partially overlapping but functionally distinct interfaces are considered: (i) glucose- and lipid-driven blood–brain barrier (BBB) dysfunction, which alters vascular permeability and exposure of CNS cells to circulating factors; (ii) the meningeal lymphatic-glymphatic-skull marrow axis, which influences inflammatory mediator clearance and immune-cell trafficking at CNS borders; and (iii) gut-brain immune and metabolic signaling, which provides recurrent microbial, low-molecular-weight metabolic, and autonomic inputs. These systems should not be regarded as independent pathways. Rather, their convergence may determine the magnitude, duration, and spatial distribution of peripheral metabolic-inflammatory signals reaching CNS border compartments and parenchymal glia.

Importantly, evidence supporting these interfaces is derived from different experimental and clinical contexts, and direct demonstration that their coordinated dysfunction causes epilepsy in MetS remains limited. We therefore consider them interacting components of a testable peripheral-to-CNS relay rather than an established linear epileptogenic cascade.

### 2.1 Glucose- and lipid-driven BBB dysfunction: vascular permeability and inflammatory exposure

The BBB consists of specialized endothelial cells connected by tight junctions, pericytes, astrocytic endfeet, basement membrane components, and associated perivascular immune cells. Under physiological conditions, this neurovascular unit limits the passage of circulating molecules and immune cells into the CNS. MetS may compromise this barrier at multiple levels through hyperglycemia, insulin resistance, dyslipidemia, oxidative stress, and hypertension.

Clinical observations provide evidence that metabolic dysfunction is associated with altered CNS barrier permeability. In a cohort including more than 1,000 individuals, diabetes mellitus was associated with an increased CSF-to-plasma albumin quotient, a clinical indicator of blood-CNS barrier dysfunction. In neurologically healthy individuals, higher body mass index and waist-to-hip ratio were also associated with subsequent increases in Qalb during long-term follow-up (Janelidze et al., 2017). Importantly, these findings demonstrate an association between metabolic abnormalities and CNS barrier dysfunction but do not establish BBB disruption as a direct cause of epilepsy.

Chronic hyperglycemia promotes the non-enzymatic glycation of proteins and lipids, leading to the accumulation of advanced glycation end-products (AGEs) (Singh et al., 2014). AGEs bind to the receptor for advanced glycation end-products (RAGE) on brain endothelial cells and activate NF-κB-dependent inflammatory signaling (Vlassara and Uribarri, 2014). This response can reduce the expression or alter the localization of tight-junction proteins, including claudin-5, occludin, and zonula occludens-1 (ZO-1), thereby increasing paracellular permeability (Stranahan et al., 2016). AGE-RAGE signaling enhances NADPH oxidase activity, oxidative stress, endothelial dysfunction, and leukocyte-adhesion molecule expression. Preclinical evidence further supports hyperglycemia-related neurovascular injury. In diabetic mouse models, chronic hyperglycemia has been associated with increased BBB permeability, diminished pericyte coverage, and reduced expression of tight-junction proteins, including claudin-5 and occludin (Rom et al., 2019). These findings provide experimental support that diabetic metabolic conditions can impair neurovascular integrity.

Barrier disruption allows extravasation of circulation-restricted molecules. Albumin is particularly relevant as it can activate TGF-β/ALK5 signaling in astrocytes upon entering the brain parenchyma (Weissberg et al., 2015). Astrocytic TGF-β signaling can promote inflammatory transcriptional programs, impair potassium and glutamate homeostasis, and contribute to epileptogenic network remodeling. Fibrinogen and other blood-derived proteins may similarly activate microglia, perivascular macrophages, and endothelial cells.

Dyslipidemia provides a complementary mechanism of BBB injury. Saturated fatty acids (SFAs) and oxidized low-density lipoprotein (oxLDL), both associated with metabolic dysfunction, activate inflammatory and oxidative pathways in endothelial and immune cells (Li et al., 2020). SFA-associated TLR4 signaling promotes NF-κB activation, cytoskeletal changes, and tight-junction instability (Nagyőszi et al., 2010). OxLDL can also induce mitochondrial dysfunction, lipid peroxidation, endothelial apoptosis, and impaired nitric oxide bioavailability. These effects may be compounded by obesity-related hypertension and microvascular dysfunction.

Once BBB integrity is compromised, circulating metabolic and inflammatory mediators and normally circulation-restricted macromolecules can gain greater access to perivascular and, under sufficiently disrupted conditions, parenchymal compartments. Resident microglia, astrocytes, and other components of the neurovascular unit may subsequently respond to these blood-derived signals. BBB dysfunction therefore acts not merely as passive leakage but as a signal-transforming interface through which systemic metabolic abnormalities can be translated into CNS inflammatory and homeostatic responses.

However, increased vascular permeability alone is unlikely to account for the persistence of CNS inflammation during chronic metabolic disease. The persistence of inflammatory signals may additionally depend on impaired clearance from CNS border compartments and altered immune-cell supply, which are discussed below in the context of the meningeal lymphatic-glymphatic-skull marrow axis.

### 2.2 The meningeal lymphatic-glymphatic-skull marrow axis: impaired clearance and border immune-cell trafficking

The meningeal lymphatic system has transformed the traditional concept of CNS immune privilege. Functional lymphatic vessels located along the dural sinuses drain CSF-derived solutes, interstitial fluid, antigens, and immune cells toward the deep cervical lymph nodes (Kollarik et al., 2025). These vessels participate in immune surveillance and clearance of inflammatory mediators from CNS border compartments.

Metabolic dysfunction may impair meningeal lymphatic function. Obesity, aging, chronic inflammation, and vascular dysfunction have been associated with reduced lymphatic drainage efficiency, altered vessel morphology, and impaired VEGF-C/VEGFR3-related lymphatic maintenance (Salvador et al., 2024; Kim and Kipnis, 2025). In the context of MetS, such abnormalities may prolong the residence of cytokines, metabolic waste products, and other inflammatory mediators within CSF and meningeal compartments, thereby extending CNS exposure beyond transient peaks of systemic inflammation.

The glymphatic system represents a related clearance pathway. Glymphatic transport facilitates CSF-interstitial fluid exchange along perivascular spaces and depends partly on aquaporin-4 (AQP4) polarization at astrocytic endfeet (Iliff et al., 2012). Diabetes, obesity, vascular injury, astrocytic reactivity, and sleep disruption have all been associated with impaired AQP4 polarization or reduced glymphatic transport (Xie et al., 2013; Chen et al., 2025). These changes may contribute to the accumulation of inflammatory mediators, lactate, damaged proteins, and metabolic by-products within the brain interstitial environment.

Within this broader metabolic and vascular context, sleep disturbance is particularly relevant to epilepsy not because it is necessarily the dominant driver of glymphatic dysfunction, but because glymphatic transport is strongly state-dependent and epilepsy itself frequently disrupts sleep architecture. Thus, sleep may act as a context-dependent amplifier of pre-existing metabolic and astrocytic clearance defects. MetS-associated sleep abnormalities and seizure-related sleep fragmentation may therefore further reduce glymphatic efficiency once the system has already been compromised by metabolic, vascular, or astrocytic dysfunction. This bidirectional interaction could contribute to the persistence of a pro-inflammatory CNS milieu, although direct evidence demonstrating that sleep-dependent glymphatic impairment mediates MetS-associated epileptogenesis remains limited.

A further component of this interface is the skull bone marrow. Direct vascular channels connecting skull marrow to the dura have been identified as routes for myeloid-cell trafficking between cranial bone marrow and CNS border tissues (Goodman et al., 2024; Liu et al., 2025a). During inflammation, neutrophils, monocytes, and other myeloid populations can migrate through these channels to the meninges without first entering the systemic circulation. This local route provides a potential source of rapidly recruited immune cells adjacent to the CNS.

MetS is associated with altered hematopoiesis and a myeloid-biased bone marrow phenotype (Cai et al., 2019). Obesity-associated inflammatory signaling can expand myeloid progenitor populations and promote the production of inflammatory monocytes and neutrophils. Recent evidence also indicates bidirectional communication between CSF and skull marrow niches, suggesting that CNS-derived inflammatory signals may influence local myelopoiesis and that skull marrow-derived cells may subsequently contribute to meningeal inflammation (Li et al., 2025).

Based on these observations, we propose a coupled clearance–immune supply model at CNS borders. MetS-associated alterations in myelopoiesis may increase the availability of inflammatory myeloid cells, whereas impairment of meningeal lymphatic and glymphatic pathways may prolong the residence of inflammatory mediators within CNS border compartments. If these processes occur concurrently, they could favor the formation of a persistent meningeal and perivascular inflammatory niche capable of sustaining glial activation.

Importantly, this model does not require direct migration of skull marrow-derived cells into the brain parenchyma. Meningeal or perivascular immune cells may influence parenchymal microglia and astrocytes indirectly through cytokines, CSF-borne mediators, vascular signaling, and secondary alterations in BBB function. In this way, the skull marrow component is proposed primarily as a regulator of border immunity rather than as a direct source of parenchymal inflammatory cells.

Direct experimental evidence linking this complete meningeal lymphatic-glymphatic-skull marrow axis to MetS-associated epileptogenesis is currently lacking. The model should therefore be considered a hypothesis-generating framework that integrates established anatomical pathways with metabolic and neuroimmune observations derived from partially distinct experimental contexts.

### 2.3 Gut-brain dysregulation: microbial metabolites, endotoxemia, and autonomic signaling

The gut-brain axis is a major source of metabolic and inflammatory signals in MetS. Obesity and insulin resistance are associated with changes in microbial composition, intestinal barrier integrity, microbial metabolite production, bile acid signaling, and vagal regulation (Cani et al., 2008; Le Chatelier et al., 2013). In contrast to the glucose- and lipid-driven vascular mechanisms discussed in Section 2.1, this section focuses primarily on gut-derived microbial products and low-molecular-weight metabolites that may modify systemic immune tone or signal to the CNS through humoral, immune, metabolic, and neural routes.

A major consequence of metabolic dysbiosis is altered production of short-chain fatty acids (SCFAs), including acetate, propionate, and butyrate. SCFAs regulate immune responses through receptors such as GPR41, GPR43, and GPR109A and can modify gene transcription through histone deacetylase inhibition (Brown et al., 2003). Butyrate-producing taxa, including members of the Faecalibacterium and Roseburia genera, are frequently reduced in obesity and MetS, although results vary across populations and dietary contexts (Cani et al., 2008). Reduced SCFA availability may weaken anti-inflammatory signaling in peripheral immune cells and alter microglial activation states after SCFAs or secondary mediators reach the CNS (Erny et al., 2015).

SCFAs are only one component of the metabolite repertoire generated or modified by the gut microbiota. Other low-molecular-weight signals potentially relevant to neuroimmune regulation include secondary bile acids, tryptophan-derived indole metabolites, and microbially influenced neurotransmitter-related compounds such as γ-aminobutyric acid (GABA). These metabolites can influence host physiology through enteroendocrine, immune, vagal, and circulating metabolic pathways, and some may indirectly modify BBB function, microglial states, or neuronal excitability. However, their roles in MetS-associated epileptogenesis remain substantially less defined than their roles in systemic metabolism or other neurological conditions. Sleep disturbance may also interact bidirectionally with gut microbial and metabolic signaling (Wang et al., 2025). MetS can also impair intestinal epithelial barrier integrity. High-fat diet exposure, oxidative stress, and dysbiosis may disrupt intestinal tight junctions and increase permeability (Erny et al., 2015). This permits the translocation of bacterial products, particularly LPS, into the circulation. Low-grade elevation of circulating LPS, often termed metabolic endotoxemia, activates TLR4-dependent signaling in peripheral monocytes, adipose tissue macrophages, endothelial cells, and potentially CNS-associated immune cells (Cani et al., 2007; Tang et al., 2025).

LPS is particularly relevant to the present framework because it may operate across several levels of peripheral-to-CNS communication. Peripherally, repeated LPS exposure can promote cytokine production and alter myeloid-cell responsiveness. Its effects on the neurovascular unit are considered here primarily as downstream consequences of a gut-derived signal rather than as a separate BBB mechanism. Repeated inflammatory exposure may also influence CNS-associated macrophages and microglia, providing biological plausibility for persistent or primed inflammatory states.

However, direct evidence that chronic metabolic endotoxemia induces canonical trained immunity in CNS microglia and thereby causes epileptogenesis remains lacking. We therefore regard LPS as a plausible recurrent conditioning signal rather than a demonstrated driver of the complete pathway.

Autonomic signaling provides another route through which gut dysfunction can influence inflammatory tone. Vagal afferents transmit visceral information to the brainstem, while efferent vagal pathways regulate immune responses through the cholinergic anti-inflammatory pathway. Obesity and MetS can impair vagal sensitivity and alter autonomic regulation of inflammation (De Lartigue et al., 2011; Pavlov and Tracey, 2012). Reduced vagal anti-inflammatory control may therefore amplify cytokine responses and worsen metabolic dysregulation.

The therapeutic relevance of the gut-brain axis is supported by ketogenic diet studies. In animal models, ketogenic diet-associated changes in gut microbial composition have been implicated in seizure protection, and microbiota transfer experiments suggest that at least part of this protection can be transferred to recipient animals (Tang et al., 2025). These findings support a functional relationship between metabolic therapy, microbial signaling, and seizure susceptibility. However, whether the same microbial pathways mediate the relationship between MetS and spontaneous epileptogenesis remains uncertain, and the relative contributions of specific taxa, SCFAs, bile-acid metabolites, tryptophan-derived compounds, GABA-related metabolites, and immune pathways require further definition.

The BBB, meningeal lymphatic-glymphatic-skull marrow axis, and gut-brain axis are anatomically and functionally distinct but potentially convergent components of peripheral-to-CNS immunometabolic communication. The BBB regulates vascular permeability and CNS exposure to circulating factors; the meningeal lymphatic-glymphatic-skull marrow axis influences inflammatory mediator clearance and immune-cell supply at CNS borders; and the gut-brain axis provides recurrent microbial, metabolic, immune, and autonomic signals.

Rather than acting independently, these interfaces may interact in a relay: gut-derived and circulating metabolic signals can challenge vascular and meningeal barriers; increased barrier permeability can enhance CNS exposure to blood-derived mediators; and impaired clearance can prolong their residence within CNS border and perivascular compartments. Together, these processes may increase the duration and intensity of metabolic-inflammatory signaling reaching microglia and astrocytes.

This integrated relay does not establish a single obligatory route to epilepsy. Rather, it defines a set of interacting interfaces that may create a permissive environment for the persistent glial reprogramming discussed in Section 3.

## 3 From metabolic exposure to inflammatory memory: microglial priming and astrocytic homeostatic failure

The dysfunction of CNS immune interfaces described in Section 2 may increase the exposure of CNS border cells and parenchymal glia to recurrent metabolic and inflammatory signals, including lipopolysaccharide (LPS), advanced glycation end-products (AGEs), oxidized lipids, saturated fatty acids, cytokines, albumin, and seizure-associated damage-associated molecular patterns (DAMPs). Chronic exposure to these signals may alter glial metabolic state, transcriptional responsiveness, and homeostatic capacity.

We conceptualize the glial component of this framework as two interacting, rather than obligatorily sequential, processes: (i) trained-immunity-like priming of microglia and CNS-associated myeloid cells, which may increase responsiveness to subsequent inflammatory or metabolic challenges; and (ii) astrocytic homeostatic failure, characterized by impaired glutamate uptake, potassium buffering, water transport, metabolic support, and extracellular-matrix regulation.

Together, these glial alterations may create a permissive environment for inhibitory-circuit dysfunction, particularly at the parvalbumin-positive interneuron-perineuronal net (PV-PNN) unit discussed in Section 4. Importantly, direct evidence linking MetS-induced glial reprogramming to selective PV-PNN dysfunction and spontaneous epileptogenesis remains limited; the following sections therefore distinguish established glial biology from mechanistic inference and testable hypotheses.

### 3.1 Microglial immunometabolic reprogramming under metabolic stress

Microglia are long-lived tissue-resident macrophages that continuously survey the CNS microenvironment. Under homeostatic conditions, they support synaptic remodeling, clearance of cellular debris, trophic signaling, and immune surveillance. Their baseline metabolism relies substantially on mitochondrial oxidative phosphorylation, although microglial metabolic states are heterogeneous and adapt to local tissue demands (Li and Barres, 2018). MetS-associated signals may shift microglia toward a metabolically activated state. LPS, AGEs, oxLDL, SFAs, hyperglycemia, extracellular ATP, and inflammatory cytokines activate pattern-recognition and metabolic receptors, including TLR4, RAGE, purinergic receptors, and cytokine receptors (Norden et al., 2016; Konttinen et al., 2019). These signals converge on NF-κB, mTOR, HIF-1α, and inflammasome-associated pathways.

In inflammatory microglial states, a shift toward greater glycolytic dependence is frequently observed. HIF-1α promotes the expression of glycolytic enzymes, including hexokinase-2 (HK2), PFKFB3, and lactate dehydrogenase A (LDHA), while mTOR signaling can reinforce this metabolic program (Cheng et al., 2014; Baik et al., 2019). Increased glycolysis may provide rapid ATP and biosynthetic intermediates required for inflammatory effector functions. However, the extent to which such glycolytic reprogramming becomes persistent or “memory-like” specifically in MetS-associated microglia remains uncertain. Chronic or recurrent metabolic activation may also be accompanied by reduced mitochondrial reserve capacity and increased reactive oxygen species (ROS), potentially limiting the return to a homeostatic state.

Metabolic stress may also promote the accumulation of lipid droplets in microglia. Lipid droplet-accumulating microglia have been described in aging, neurodegeneration, and obesity-related models (Marschallinger et al., 2020; Loving et al., 2021). These cells may exhibit altered lysosomal function, mitochondrial impairment, elevated ROS production, pro-inflammatory cytokine release, and complement-associated synaptic remodeling (Shimabukuro et al., 2016; Krasemann et al., 2017). Although lipid droplets may initially serve adaptive lipid-buffering functions, persistent accumulation can also reflect impaired lipid handling. Whether this phenotype represents a stable component of MetS-associated microglial priming in epileptogenesis remains unknown.

Taken together, these observations suggest that microglial metabolic reprogramming may do more than accompany inflammation; it may influence the magnitude, duration, and responsiveness of subsequent inflammatory states. However, whether these metabolic changes persist after normalization of the MetS environment and fulfill criteria for trained immunity must be established experimentally.

### 3.2 From transient activation to persistent sensitization: a trained-immunity-like model

#### 3.2.1 Beyond acute microglial responses

Conventional descriptions of neuroinflammation emphasize the immediate activation of microglia following injury, infection, or cytokine exposure. In this framework, microglial responses are often viewed as stimulus-dependent and reversible once the initiating signal is removed (Block et al., 2007). However, accumulating evidence indicates that prior inflammatory or metabolic insults can durably alter microglial responsiveness long after the original stimulus has subsided (Perry and Holmes, 2014; Niraula et al., 2017). This persistence cannot be fully explained by ongoing tissue damage alone. The concept of trained immunity provides a mechanistic framework for understanding such inflammatory memory. Trained immunity refers to antigen-independent innate immune memory in which a primary stimulus induces sustained metabolic and epigenetic changes that enhance responses to later, unrelated challenges (Netea et al., 2011, 2016). This phenomenon is well-characterized in peripheral monocytes and macrophages exposed to β-glucan, BCG vaccination, oxidized lipoproteins, and Western-diet stimuli (Kleinnijenhuis et al., 2012; Quintin et al., 2012; Arts et al., 2018). The canonical program involves: (i) metabolic reprogramming toward glycolysis via mTOR/HIF-1α (Cheng et al., 2014; Saeed et al., 2014); (ii) accumulation of immunoregulatory metabolites (Arts et al., 2016; Bekkering et al., 2018); (iii) epigenetic remodeling at inflammatory gene loci (Foster et al., 2007; Ostuni et al., 2016); and (iv) enhanced cytokine/ROS responses upon re-stimulation (Novakovic et al., 2016). Whether microglia in MetS-associated epileptogenesis undergo canonical trained immunity remains unresolved. Direct evidence for persistent chromatin remodeling, trained metabolic flux, and enhanced recall responses in microglia within MetS-related epilepsy models is currently lacking. Therefore, we use the term trained-immunity-like microglial priming to denote a plausible state of persistent immunometabolic sensitization requiring experimental validation.

#### 3.2.2 Evidence supporting persistent microglial priming in the CNS

Although not fully validated in MetS-epilepsy, substantial evidence supports long-lasting microglial priming in other CNS contexts. Systemic LPS, infection, or trauma can durably alter microglial transcription, morphology, and cytokine responsiveness weeks after peripheral stimulation ceases (Fenn et al., 2012; Wendeln et al., 2018). Primed microglia exhibit exaggerated inflammatory responses to subsequent CNS insults, including ischemia, injury, and neurodegeneration (Delpech et al., 2019; Woodburn et al., 2021). A landmark study demonstrated that systemic inflammation induces innate immune memory in the brain, accelerating amyloid pathology in Alzheimer’s models through microglial chromatin remodeling (Wendeln et al., 2018). Aging-associated microglial priming involves lipid droplet accumulation, mitochondrial stress, and enhanced IL-1β responsiveness (Raj et al., 2014; Spittau, 2017). Recent single-cell analyses reveal disease-associated microglial states with metabolic reprogramming and inflammasome activation (Keren-Shaul et al., 2017; Krasemann et al., 2017; Sala Frigerio et al., 2019). These observations establish that persistent and metabolically reprogrammed microglial states are biologically possible within the CNS.

However, they should be regarded as supporting biological plausibility rather than direct evidence that MetS induces an equivalent state during epileptogenesis.

#### 3.2.3 MetS as a myeloid training signal source

MetS provides recurrent training stimuli: chronic endotoxemia (intestinal permeability), saturated fatty acids, oxLDL, AGEs, hyperglycemia-related oxidative stress, and altered adipokines (Hotamisligil, 2017; Rohm et al., 2022). Western-diet exposure has been shown to induce NLRP3-dependent reprogramming of bone marrow myeloid progenitors, with persistent inflammatory and epigenetic changes that can outlast dietary normalization (Bekkering et al., 2016; Christ et al., 2018). OxLDL induces trained monocyte phenotypes with enhanced cytokines, glycolysis, and histone modifications (Bekkering et al., 2014). These findings establish MetS-related dietary and lipid signals as bona fide peripheral training stimuli. However, whether the same trained phenotype is generated specifically within cranial bone marrow and whether such cells subsequently alter seizure susceptibility remain unknown. Myeloid cells from cranial marrow can access meningeal and perivascular compartments through skull marrow–meningeal routes (Herisson et al., 2018; Cugurra et al., 2021). Whether MetS-conditioned or trained myeloid populations preferentially use these pathways remains unknown. Such border-resident or recruited cells could nevertheless influence parenchymal microglia through CSF-derived cytokines and vascular signals without requiring extensive parenchymal infiltration (Mrdjen et al., 2018; Prinz et al., 2019).

#### 3.2.4 A testable mechanistic bridge

We propose trained-immunity-like microglial priming as a testable mechanistic bridge between chronic MetS and glial dysfunction. Repeated metabolic danger signals may shift microglia toward a primed state with: (i) enhanced glycolysis and reduced mitochondrial reserve; (ii) increased mTOR/HIF-1α and NLRP3 signaling; (iii) accumulation of succinate/fumarate; and (iv) hypothesized (undemonstrated) chromatin remodeling at inflammatory loci. Under this model, subsequent challenges—including seizures, infection, recurrent BBB dysfunction, or tissue-derived DAMPs—could evoke disproportionately large cytokine, ROS, complement, or protease responses. Such amplified responses could secondarily impair astrocytic glutamate uptake, potassium buffering, and extracellular-matrix integrity (Vezzani et al., 2011; Vezzani and Viviani, 2015). Importantly, the proposed primed state is conceptualized as a modifier of seizure susceptibility rather than a sufficient cause of epilepsy.

#### 3.2.5 Testable predictions and evidence gaps

If trained-immunity-like microglial priming contributes to MetS-associated epileptogenesis, the framework generates several falsifiable predictions:

1. Durable metabolic reprogramming. Microglia isolated from MetS-exposed animals should retain altered glycolytic flux, TCA-cycle metabolites, or mitochondrial reserve after normalization of the peripheral metabolic environment.
2. Persistent epigenetic remodeling. Chromatin profiling should reveal sustained enrichment of activating marks such as H3K4me3 or H3K27ac at inflammatory loci, including IL-1β, TNF-α, IL-6, or NLRP3, after removal of the initial metabolic stimulus.
3. Enhanced recall responses. Microglia from previously MetS-exposed animals should show exaggerated cytokine or ROS production upon standardized secondary stimulation after a recovery period.
4. Interventional reversibility. Pharmacological or genetic interruption of mTOR, HIF-1α, NLRP3, or related metabolic-epigenetic pathways during MetS exposure should attenuate subsequent inflammatory recall responses and reduce seizure susceptibility.
5. Functional necessity. Microglial depletion or cell-specific disruption of candidate pathways should reduce later epileptogenic susceptibility if microglial priming is mechanistically required.
6. Peripheral-to-CNS transfer. Bone marrow transplantation, parabiosis, or adoptive-transfer approaches could test whether MetS-conditioned hematopoietic cells are sufficient to transmit part of the neuroinflammatory or seizure-susceptibility phenotype.

None of these predictions has yet been systematically validated in MetS-associated epilepsy. Accordingly, the value of the framework lies in generating experimentally discriminable hypotheses rather than asserting that the entire pathway has already been established.

#### 3.2.6 Therapeutic implications

Improvement in systemic metabolic parameters may reduce ongoing inflammatory and metabolic stimulation, but the extent and timing of CNS immune normalization are poorly defined. In peripheral trained-immunity models, epigenetic remodeling, altered progenitor composition, and metabolic adaptations can persist after withdrawal of the initiating stimulus (Christ et al., 2018; Mitroulis et al., 2018). Whether comparable persistence occurs in CNS microglia after metabolic correction remains unknown.

Therefore, incomplete seizure improvement after metabolic intervention should not be interpreted as evidence for persistent trained immunity by itself. Nevertheless, the possibility of delayed reversal of immunometabolic changes provides a rationale for testing whether earlier metabolic intervention has greater disease-modifying potential than intervention after chronic neuroinflammatory and circuit remodeling have become established.

Pharmacological modulation of NLRP3, mTOR, HIF-1α, mitochondrial function, or related pathways may provide useful experimental tools for testing this model. At present, such interventions should be regarded primarily as mechanistic probes or future therapeutic candidates rather than established treatments for MetS-associated epilepsy. These proposed features are summarized schematically in Figure 2.

**Figure 2:**
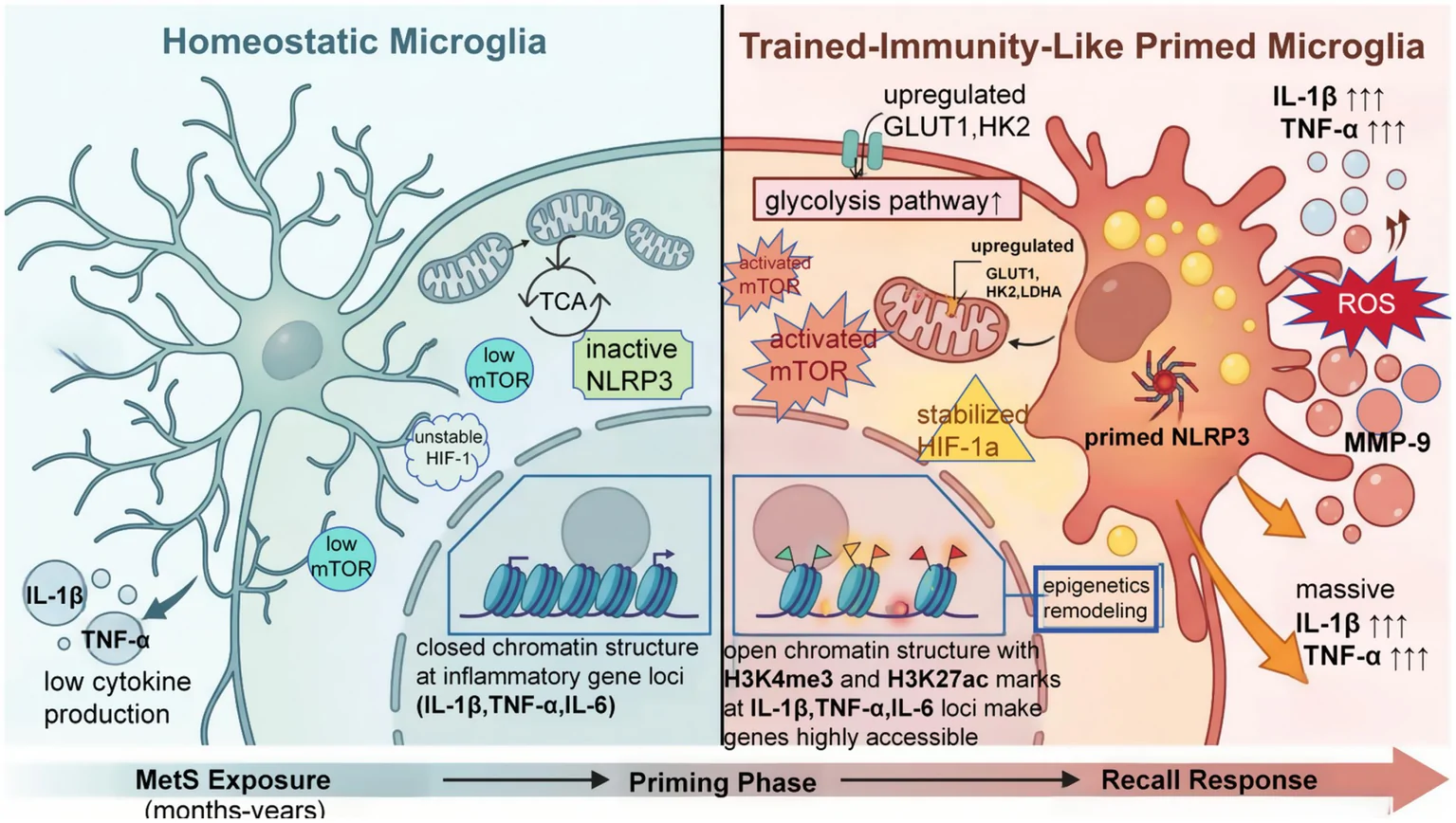
Candidate metabolic and epigenetic features of trained-immunity-like microglial priming.

The schematic contrasts a homeostatic reference state (left) with a hypothesized primed state (right), rather than defining discrete or universally observed microglial phenotypes. Candidate features include altered glycolytic activity and associated proteins (GLUT1, HK2, and LDHA), changes in tricarboxylic acid-cycle metabolites such as succinate and fumarate, lipid droplet accumulation, altered mTOR/HIF-1α signaling, NLRP3 inflammasome priming, and persistent chromatin changes, potentially involving H3K4me3 or H3K27ac enrichment at inflammatory loci. These features have not been established as a coordinated, persistent program in MetS-associated epileptogenesis. The model predicts enhanced cytokine, ROS, or protease responses to a standardized secondary challenge after withdrawal of the initial exposure and a recovery period. Such responses must be distinguished from ongoing activation and immune tolerance. The timeline is conceptual and illustrates exposure, recovery, and rechallenge rather than an established disease trajectory.

### 3.3 Astrocytic homeostatic failure

Astrocytes translate immune and metabolic signals into changes in neuronal homeostasis, regulating extracellular glutamate, potassium, water movement, energy substrate availability, redox balance, vascular coupling, and extracellular-matrix composition (Allen and Lyons, 2018; Verkhratsky and Nedergaard, 2018). These functions make astrocytes critical intermediaries between microglial inflammatory memory and circuit dysfunction. Chronic exposure to cytokines IL-1β, TNF-α, IL-6, albumin (TGF-β/ALK5 signaling), ROS, AGEs, and metabolic stress induces astrocytic reactivity (Ransohoff, 2016; Liddelow et al., 2017). Reactive astrocytes display marked heterogeneity; simple binary classifications are inadequate (Zamanian et al., 2012; Escartin et al., 2021). Nevertheless, several homeostatic functions are repeatedly disrupted in inflammatory/epileptogenic settings:

#### 3.3.1 Impaired glutamate uptake

Reduced EAAT1 and EAAT2/GLT-1 expression/function decreases astrocytic glutamate clearance (Rothstein et al., 1996; Proper et al., 2002). Increased extracellular glutamate promotes excitotoxicity and synchronized firing (Hubbard et al., 2018). PV+ interneurons receive substantial glutamatergic input and may be particularly vulnerable to sustained excitatory and metabolic stress; however, direct evidence that MetS-related EAAT dysfunction selectively injures PV+ interneurons remains limited (Kann et al., 2014).

#### 3.3.2 Compromised potassium buffering

Reduced Kir4.1 expression or spatial disorganization impairs K + clearance (Djukic et al., 2007; Heuser et al., 2012). Elevated extracellular K^+^ depolarizes neuronal membranes and can reduce Na^+^-channel availability, while disturbances in K^+^ gradients may also influence chloride homeostasis through KCC2-dependent coupling, potentially reducing inhibitory efficacy (Kofuji and Newman, 2004). Because fast-spiking PV+ interneurons operate within narrow ionic and energetic constraints, impaired potassium homeostasis is mechanistically relevant to their function, although selective PV vulnerability to MetS-induced Kir4.1 dysfunction has not been directly established.

#### 3.3.3 Disturbed AQP4 polarization

AQP4 mislocalization at astrocytic endfeet impairs water flux and glymphatic clearance (Iliff et al., 2012), mechanistically linking astrocytic dysfunction to Section 2 clearance defects. Perivascular inflammation, BBB leakage, sleep disruption, and recurrent seizures exacerbate AQP4 disruption (Iliff et al., 2012; Xie et al., 2013). In this context, AQP4 dysfunction should be viewed as a shared amplifier of impaired fluid and ionic homeostasis rather than a MetS-specific epileptogenic mechanism.

#### 3.3.4 Inadequate metabolic/trophic support

Astrocytes regulate glycogen metabolism and supply lactate to neurons via monocarboxylate transporters (Magistretti and Allaman, 2015; Bonvento and Bolaños, 2021). Metabolic dysfunction, mitochondrial stress, and inflammation reduce this support, increasing vulnerability of high-energy-demand neurons (Inan et al., 2016). Astrocytes also influence BDNF, GDNF, and neuregulin signaling, though directionality depends on disease stage/model (Fulmer et al., 2014; Xenos et al., 2018). Accordingly, impaired astrocytic metabolic support is a biologically plausible contributor to PV+ interneuron vulnerability, but should not be interpreted as a demonstrated MetS-specific mechanism.

#### 3.3.5 Extracellular-matrix dysregulation

Cytokine-driven signaling increases matrix metalloproteinase release, alters chondroitin sulfate proteoglycan composition, and interacts with complement-mediated synaptic remodeling (Dubey et al., 2017; Fawcett et al., 2019). These changes can destabilize perineuronal nets (PNNs), which are particularly enriched around PV+ interneurons (Cabungcal et al., 2013; Wen et al., 2018). This provides a plausible structural route through which glial inflammation could influence the PV-PNN unit, although direct evidence for MetS-driven PNN degradation as a cause of epileptogenesis remains limited.

### 3.4 A glial bridge from metabolic stress to inhibitory-circuit vulnerability

Microglial trained-immunity-like priming and astrocytic homeostatic failure may represent complementary components of an altered glial environment. Primed or persistently reactive microglia can produce increased amounts of IL-1β, TNF-α, IL-6, ROS, nitric oxide, complement components, and extracellular proteases, whereas dysfunctional astrocytes may fail to adequately maintain glutamate, potassium, redox, metabolic, and extracellular-matrix homeostasis (Wendeln et al., 2018; Matejuk and Ransohoff, 2020; Netea et al., 2020; Sofroniew, 2020).

The resulting environment would not necessarily affect all neuronal populations equally. Neurons with high energetic demands, narrow ionic operating ranges, and dependence on specialized extracellular-matrix structures would be expected to be particularly vulnerable to combined metabolic, oxidative, and homeostatic stress.

PV+ fast-spiking interneurons satisfy several of these vulnerability criteria. Their sustained high-frequency firing requires substantial mitochondrial and Na^+^/K^+^-ATPase capacity; their firing precision depends on tightly regulated extracellular ionic conditions; and many PV+ interneurons are surrounded by PNNs that contribute to synaptic stability and protection against oxidative stress (Sohal et al., 2009; Cabungcal et al., 2013; Hu et al., 2014).

We therefore propose the PV-PNN unit as a candidate circuit-level convergence point for immunometabolic injury rather than as an exclusively or definitively established target of MetS.

Under this framework, persistent glial dysfunction may lower the resilience of PV-mediated inhibition, thereby increasing susceptibility to excitation-inhibition imbalance when additional stressors occur. Direct experimental demonstration that MetS selectively impairs the PV-PNN unit before the onset of spontaneous seizures remains an important unresolved prediction of the model.

## 4 The PV-PNN unit as a circuit-level convergence point of immunometabolic stress

The glial alterations described in Section 3 may not affect all neuronal populations equally. Among cortical inhibitory neurons, parvalbumin-positive (PV+) fast-spiking interneurons represent a plausible candidate vulnerable population because of their high metabolic demand, rapid firing properties, and dependence on tightly regulated ionic and extracellular-matrix conditions. These cells provide powerful perisomatic inhibition to pyramidal neurons and are essential for feedforward and feedback inhibition, gamma-frequency synchronization, and network stability (Sohal et al., 2009; Hu et al., 2014).

We therefore propose the PV interneuron-perineuronal net (PV-PNN) unit as a candidate circuit-level convergence point at which immunometabolic and glial disturbances may be translated into impaired inhibitory control. This proposal does not imply that PV+ interneurons are uniquely or universally affected in MetS; rather, it identifies a biologically plausible neuronal population whose physiological properties make it especially suitable for mechanistic testing.

### 4.1 Physiological features underlying candidate PV-PNN vulnerability

High energy demand and metabolic vulnerability. PV+ interneurons sustain high-frequency firing and therefore require substantial mitochondrial capacity and continuous energy production to support Na^+^/K^+^-ATPase activity, membrane repolarization, and calcium handling (Kann et al., 2014; Inan et al., 2016). Parvalbumin protein provides rapid intracellular calcium buffering critical for repetitive firing (Schwaller, 2012). These physiological characteristics may make PV+ interneurons particularly sensitive to reductions in metabolic substrate availability, mitochondrial dysfunction, and oxidative stress.

Structural protection by perineuronal nets. Most PV+ interneurons are surrounded by PNNs composed of hyaluronan, chondroitin sulfate proteoglycans (particularly aggrecan), link proteins, and tenascin-R (Sorg et al., 2016; Fawcett et al., 2019). PNNs contribute to synaptic stabilization, regulation of extracellular ionic and molecular diffusion, restriction of excessive plasticity, and protection against oxidative stress. Loss or remodeling of PNN integrity may therefore remove an important layer of structural and biochemical protection around PV+ interneurons, potentially increasing their vulnerability to concurrent metabolic, ionic, and inflammatory stress.

### 4.2 Multi-hit convergence on the PV-PNN unit

We propose six interacting classes of stress through which MetS-associated glial and metabolic dysfunction could converge on the PV-PNN unit. These mechanisms are grouped into metabolic/oxidative, homeostatic, and structural categories. They should be regarded as a mechanistic synthesis derived from partially overlapping experimental literatures rather than as six sequentially demonstrated events in MetS-associated epilepsy.

#### 4.2.1 Metabolic and oxidative insults

##### 4.2.1.1 Bioenergetic substrate deprivation

Inflammatory astrocytic states can be associated with impaired mitochondrial function and altered metabolic support via the astrocyte-neuron lactate shuttle (Magistretti and Allaman, 2015; Bonvento and Bolaños, 2021). MetS-associated vascular and metabolic abnormalities can impair cerebral microvascular function and substrate delivery through insulin resistance, endothelial dysfunction, and BBB disruption (Zlokovic, 2011). Reduced metabolic support could compromise Na^+^/K^+^-ATPase activity, calcium extrusion, and action-potential recovery, potentially imposing a disproportionate burden on high-firing PV+ interneurons.

##### 4.2.1.2 Oxidative stress and parvalbumin dysfunction

Microglial activation increases ROS and reactive nitrogen species through NADPH oxidase, inducible nitric oxide synthase, and mitochondrial dysfunction (Brown and Neher, 2014; Simpson and Oliver, 2020). Reactive astrocytic states may provide reduced antioxidant support in some inflammatory contexts. Oxidative stress damages mitochondrial proteins, membrane transporters, and parvalbumin itself (Cabungcal et al., 2013). Reduced calcium-buffering efficiency compromises repetitive firing. Calcium overload further increases mitochondrial ROS and activates cell-injury pathways. PNN components are also vulnerable to oxidative modification, increasing susceptibility to proteolytic degradation.

#### 4.2.2 Homeostatic insults

##### 4.2.2.1 Loss of trophic support

PV+ interneurons depend on BDNF/TrkB and neuregulin-1/ErbB4 signaling for maturation, synaptic connectivity, PV expression, and firing properties (Fazzari et al., 2010; Xenos et al., 2018). Chronic microglial and astrocytic activation disrupts trophic factor availability and signaling. Reduced trophic support decreases PV expression, weakens perisomatic synapses, and impairs inhibitory terminal maintenance. These changes could progressively weaken inhibitory-circuit stability.

##### 4.2.2.2 Extracellular potassium accumulation

Astrocytes regulate extracellular potassium via Kir4.1 channels. In inflammatory and epileptic conditions, reduced Kir4.1 expression impairs potassium uptake (Djukic et al., 2007; Heuser et al., 2012). Elevated extracellular K^+^ depolarizes neuronal membranes and can reduce Na^+^-channel availability, potentially compromising repetitive firing under sustained network activity. Because chloride homeostasis is partly coupled to transmembrane K^+^ gradients through KCC2, marked disturbances in extracellular K^+^ may also influence the efficacy of GABAergic inhibition. These effects provide a plausible route by which impaired astrocytic K^+^ buffering could destabilize inhibitory network function, although preferential impairment of PV+ interneurons remains to be demonstrated.

##### 4.2.2.3 Glutamate accumulation

Astrocytic EAAT1/EAAT2 dysfunction reduces glutamate clearance (Rothstein et al., 1996; Proper et al., 2002). PV+ interneurons receive substantial glutamatergic input. Increased extracellular glutamate overstimulates NMDA and AMPA receptors, producing calcium influx, mitochondrial stress, and excitotoxic injury. Glutamate accumulation simultaneously impairs PV+ cells and enhances pyramidal neuron excitation. Because PV+ interneurons receive substantial excitatory input required to recruit rapid feedback inhibition, excessive glutamatergic signaling could simultaneously increase their metabolic burden while enhancing pyramidal-cell excitation. This dual effect provides a plausible mechanism for weakening inhibitory control without requiring selective PV neuronal death.

#### 4.2.3 Structural insult

##### 4.2.3.1 Proteolytic PNN remodeling

Inflammatory microglia, reactive astrocytes, and infiltrating myeloid cells increase MMP-9, ADAMTS proteases, and complement activity (Dubey et al., 2017; Levy et al., 2022). These systems degrade aggrecan, brevican, and hyaluronan-associated PNN structures. Loss of PNN integrity destabilizes perisomatic synapses, alters extracellular ion and neurotransmitter diffusion, increases structural plasticity, and removes local protection against oxidative and inflammatory stress (Wen et al., 2018; Crapser et al., 2020). MMP-9 is of particular interest because it may provide a molecular link between neurovascular extracellular-matrix remodeling and PNN disruption.

### 4.3 Synergistic amplification

The six insults do not occur in fixed sequence but interact continuously. Energy failure reduces capacity to remove calcium and maintain ion gradients. Calcium dysregulation increases mitochondrial ROS production. Oxidative stress damages parvalbumin, mitochondrial proteins, and PNN components. PNN degradation increases PV+ cell exposure to ionic, oxidative, and inflammatory stress. Potassium accumulation increases energetic cost of firing and promotes sodium-channel inactivation. Glutamate accumulation drives calcium influx and recurrent excitation. Trophic withdrawal reduces synaptic and metabolic resilience. This creates a multi-hit vulnerability state. A single disturbance may be insufficient to produce a detectable circuit phenotype, whereas the convergence of several moderate insults could progressively impair PV-mediated inhibition and lower the threshold for pathological synchronization.

### 4.4 From PV-PNN dysfunction to E/I imbalance

PV+ interneuron impairment, due to reduced feedforward and feedback inhibition and disrupted gamma-band synchronization (Cardin et al., 2009; Buzsáki and Wang, 2012; Veit et al., 2017), along with loss of PNN integrity increasing structural plasticity and reducing perisomatic synapse stability, shifts the E/I balance toward excitation. Network-level consequences include reduced perisomatic inhibition amplitude and precision, impaired suppression of recurrent pyramidal excitation, increased extracellular potassium and glutamate, abnormal gamma oscillations, enhanced neuronal synchrony, and lower seizure threshold. The framework therefore proposes a circuit-level bridge in which metabolic and inflammatory stress may promote trained-immunity-like microglial priming and astrocytic dysfunction, which could converge on the PV-PNN unit, weaken perisomatic inhibition, and increase susceptibility to network hyperexcitability. The complete sequence remains to be demonstrated in MetS-specific models (Figure 3).

**Figure 3:**
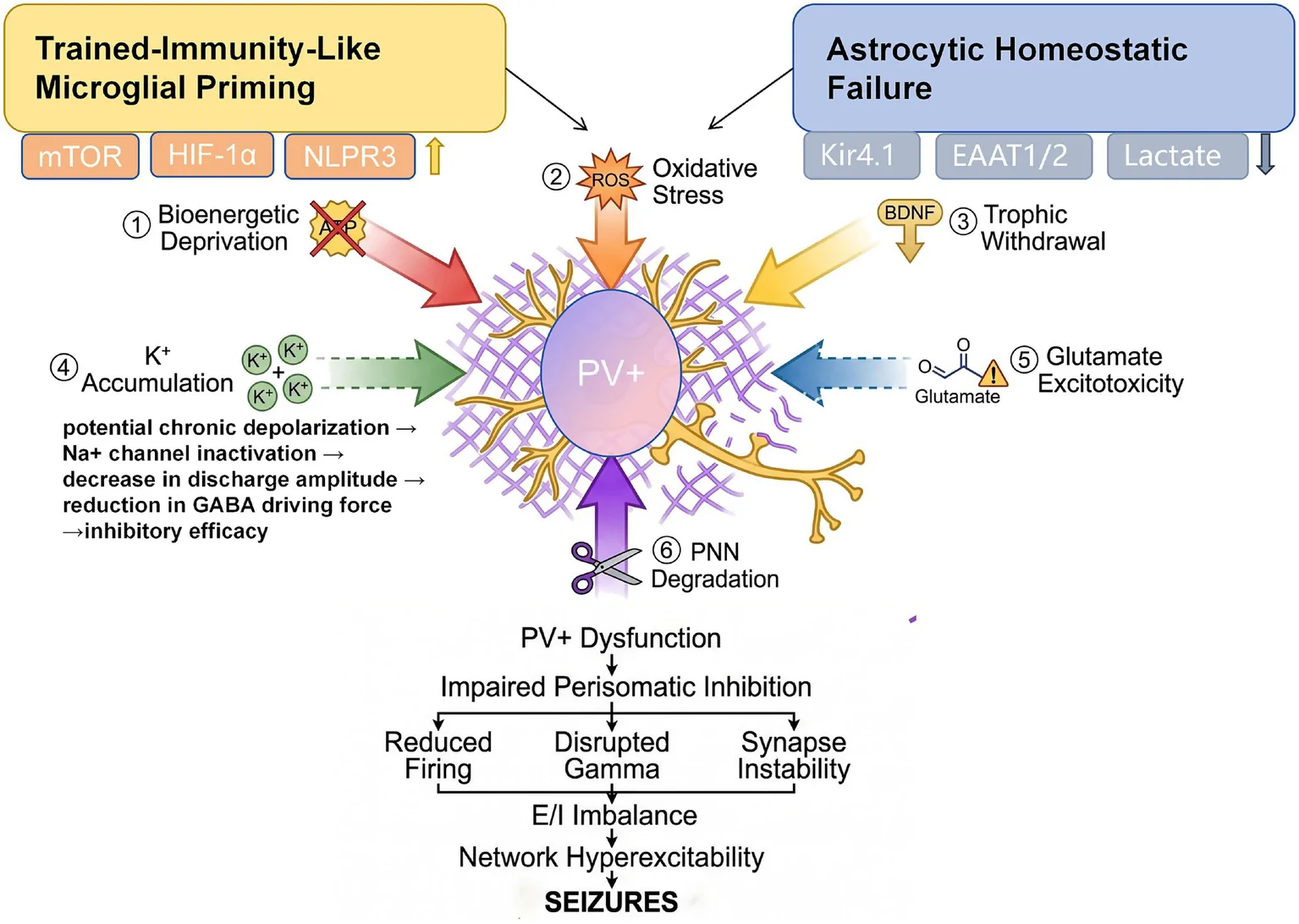
Proposed multi-hit convergence on the parvalbumin-positive interneuron–perineuronal net (PV-PNN) unit.

Six potentially interacting stress pathways are proposed: (1) altered energy-substrate supply or utilization; (2) oxidative and nitrosative stress; (3) altered trophic signaling; (4) extracellular K^+^ accumulation associated with impaired astrocytic buffering; (5) excess glutamatergic signaling associated with impaired uptake; and (6) PNN remodeling through protease-dependent and immune-associated processes. Microglial and astrocytic changes may contribute to these pathways through overlapping mechanisms, without an obligatory temporal sequence. Their combined effects could impair PV-interneuron firing precision and perisomatic inhibition, potentially increasing susceptibility to pathological network activity. Selective PV-PNN injury, synergistic interactions among the proposed insults, and progression to spontaneous epilepsy remain to be demonstrated in MetS-specific models. Changes in PV expression or PNN labeling alone do not establish neuronal loss or inhibitory failure, and network consequences may vary with brain region and disease stage.

## 5 Therapeutic implications

The framework described above contains both potentially reversible functional abnormalities and more persistent structural changes. Metabolic, inflammatory, and ionic disturbances may be amenable to correction, whereas prolonged extracellular-matrix remodeling, synaptic reorganization, or interneuron dysfunction may be less readily reversible. This distinction raises the possibility that the timing of intervention could influence therapeutic efficacy.

Potential strategies include correction of systemic metabolic abnormalities, modulation of inflammatory signaling, restoration of astrocytic ionic and metabolic homeostasis, protection of extracellular-matrix integrity, and preservation of inhibitory-circuit function. However, most of these approaches have not been tested specifically in MetS-associated epilepsy. Accordingly, the therapeutic discussion below is intended primarily to identify experimentally tractable pathway nodes rather than to recommend current clinical treatment.

### 5.1 Candidate molecular hub nodes: mechanistic rationale and translational readiness

Five candidate molecular hub nodes—NLRP3, mTOR, HIF-1α, MMP-9, and Kir4.1—were prioritized because they occupy complementary convergence points within the proposed immunometabolic synaptopathy framework. Rather than being specific to MetS, these molecules participate in interconnected pathways involving metabolic stress, innate immune activation, immunometabolic reprogramming, astrocytic homeostasis, neurovascular dysfunction, extracellular-matrix remodeling, and neuronal excitability (Djukic et al., 2007; Cheng et al., 2014; Vafadari et al., 2016; Christ et al., 2018; Swanson et al., 2019). The nature and strength of evidence differ substantially among these targets. mTOR has direct clinical validation in a genetically defined epilepsy—tuberous sclerosis complex (TSC)—where adjunctive everolimus reduced treatment-resistant focal-onset seizures in the randomized Phase III EXIST-3 trial (French et al., 2016). By contrast, NLRP3 and MMP-9 are supported primarily by mechanistic studies of innate inflammation, neuroinflammation, and experimental epileptogenesis (Wilczynski et al., 2008; Swanson et al., 2019). HIF-1α has strong immunometabolic rationale and preclinical epilepsy-related evidence, whereas Kir4.1 is supported by genetic, experimental, and human neuropathological evidence linking impaired astrocytic K^+^ homeostasis to seizure susceptibility (Djukic et al., 2007; Bockenhauer et al., 2009; Heuser et al., 2012; Fu et al., 2021).

Importantly, evidence supporting a molecular target in epilepsy, metabolic disease, or neuroinflammation should not be interpreted as direct validation of that target in MetS-associated epileptogenesis. For example, Western-diet exposure induces NLRP3-dependent reprogramming of bone-marrow innate immune cells (Christ et al., 2018), but it remains unknown whether an equivalent process in cranial or systemic myeloid compartments causally promotes glial reprogramming, PV-PNN dysfunction, and spontaneous epilepsy in MetS. Similarly, the clinical efficacy of mTOR inhibition in TSC provides proof of targetability in an mTOR-driven epilepsy but does not establish therapeutic efficacy in metabolically associated epileptogenesis (French et al., 2016).

Mechanistically, NLRP3, mTOR, and HIF-1α can be viewed as an interconnected upstream immunometabolic module. NLRP3 couples cellular danger sensing to caspase-1-dependent maturation of IL-1β and IL-18 (Swanson et al., 2019), whereas mTOR and HIF-1α coordinate nutrient-sensitive glycolytic and inflammatory programs central to trained-immunity biology (Cheng et al., 2014). Metabolic intermediates can further reinforce this interaction; for example, succinate stabilizes HIF-1α and promotes IL-1β expression in activated innate immune cells (Tannahill et al., 2013). Western-diet-induced NLRP3-dependent innate immune reprogramming provides additional evidence that metabolic and inflammatory pathways can remain functionally coupled after chronic dietary exposure (Christ et al., 2018).

MMP-9 and Kir4.1 occupy more downstream positions within the proposed framework and are particularly relevant to the transition from glial and neurovascular dysfunction to circuit instability. MMP-9 contributes to extracellular-matrix remodeling and has been implicated experimentally in epileptogenesis (Wilczynski et al., 2008; Vafadari et al., 2016). Kir4.1, encoded by KCNJ10, is a major determinant of astrocytic extracellular K^+^ buffering; conditional Kir4.1 loss disrupts potassium and glutamate uptake (Djukic et al., 2007), reduced perivascular Kir4.1 expression has been reported in human mesial temporal lobe epilepsy (Heuser et al., 2012), and pathogenic KCNJ10 variants cause EAST/SeSAME syndrome with epilepsy (Bockenhauer et al., 2009).

Pharmacological tractability should not be equated with immediate clinical repurposability. Several compounds previously linked to these hubs are indirect modulators or have substantial pharmacokinetic and safety limitations. Glyburide (glibenclamide), for example, inhibits NLRP3-associated signaling experimentally but shows negligible penetration across the intact human BBB and carries clinically relevant hypoglycemia risk (Clemens et al., 2015; Marie et al., 2019). Metformin has pleiotropic effects on mitochondrial complex I, AMPK/mTOR-related metabolism, and HIF-1α-associated signaling rather than functioning as a selective HIF-1α inhibitor (Wheaton et al., 2014). Likewise, doxycycline and minocycline exert broad anti-inflammatory and matrix-metalloproteinase-modulating effects and should not be regarded as selective MMP-9 inhibitors (Tikka et al., 2001; Vandenbroucke and Libert, 2014).

Nevertheless, several emerging findings strengthen the experimental tractability of these nodes. In the MiTS randomized placebo-controlled trial, metformin was associated with a greater reduction in seizure frequency than placebo as a secondary outcome in TSC; however, only 21 participants contributed to the seizure analysis, and the result should therefore be regarded as exploratory (Amin et al., 2021). Metformin has also shown beneficial neurological effects in Lafora disease mouse models, while a small observational cohort suggested slower clinical progression among treated patients (Burgos et al., 2023). These findings broaden the epilepsy-related translational context of metformin but do not establish efficacy in MetS-associated epilepsy.

More target-specific tools are also emerging. The CNS-penetrant NLRP3 inhibitor NT-0796 demonstrated substantial CSF exposure and reductions in central and systemic inflammatory biomarkers, including CSF IL-1β, during a 28-day Phase 1b study in Parkinson disease (Clarke et al., 2025). These findings provide human evidence that pharmacological NLRP3 inhibition can achieve CNS target engagement, although they provide no evidence of antiseizure efficacy. Finally, the small molecule 2-D08 was recently identified as a Kir4.1 activator and reduced demyelination in mouse and marmoset models of experimental autoimmune encephalomyelitis (Liu et al., 2025b). Because these effects were characterized primarily in oligodendrocyte-lineage cells and demyelinating disease models, 2-D08 should be considered evidence of Kir4.1 pharmacological tractability rather than an established epilepsy-directed therapy.

Accordingly, these five nodes should be regarded as experimentally tractable points within the proposed network rather than as equivalent or clinically validated therapeutic targets. Their principal current value lies in enabling pathway-specific testing of the immunometabolic synaptopathy framework, while future disease-specific studies will be required to determine whether any can support prevention or disease modification in MetS-associated epilepsy. Representative tools, current translational status, and major limitations are summarized in Table 1.

**Table 1:** Candidate molecular hub nodes within the proposed immunometabolic synaptopathy framework and their current translational status.

Candidate hub	Mechanistic position	Representative tools/current status	Major limitation for MetS-associated epilepsy
NLRP3	Metabolic danger sensing; inflammasome activation; IL-1β/IL-18 maturation; peripheral trained-immunity signaling (Christ et al., 2018; Swanson et al., 2019)	MCC950—potent experimental inhibitor; rheumatoid-arthritis clinical development was discontinued because of hepatotoxicity. Dapansutrile—clinical-stage inhibitor. NT-0796—CNS-penetrant inhibitor with Phase 1b human CNS target-engagement data (Clarke et al., 2025). Selnoflast—CNS-oriented clinical-stage inhibitor. Glyburide—experimental NLRP3-modulating activity.	Direct NLRP3-dependent causation in MetS-associated epilepsy is unproven. Compound-specific limitations include CNS penetration and safety; glyburide has poor BBB penetration and hypoglycemia risk (Clemens et al., 2015; Marie et al., 2019).
mTOR	Nutrient/insulin sensing; glycolytic reprogramming; interaction with HIF-1α and inflammatory pathways (Cheng et al., 2014)	Rapamycin/sirolimus; everolimus clinically reduces TSC-associated treatment-resistant seizures (French et al., 2016).	Clinical antiseizure efficacy is established mainly in mTOR-driven genetic epilepsy and cannot be directly extrapolated to MetS-associated epilepsy.
HIF-1α	Glycolytic reprogramming; pseudohypoxic signaling; inflammatory metabolic adaptation	miR-221-3p-mediated HIF-1α suppression reduces microglial activation and seizures preclinically (Fu et al., 2021). Metformin indirectly modulates HIF-1α-related metabolism; exploratory seizure data exist in TSC and Lafora disease (Amin et al., 2021; Burgos et al., 2023).	No selective, clinically validated CNS HIF-1α inhibitor for epilepsy; metformin should not be considered a selective HIF-1α-targeted therapy.
MMP-9	BBB/ECM remodeling; PNN degradation; potential vascular-to-PV-PNN bridge	Doxycycline/minocycline have broad MMP-modulating effects; selective MMP-9 inhibitors remain investigational (Vandenbroucke and Libert, 2014)	Clinically available drugs are nonspecific; disease-modifying efficacy in epilepsy and MetS-associated epileptogenesis is unestablished.
Kir4.1/KCNJ10	Astrocytic K^+^ buffering; extracellular ionic homeostasis; link between astrocytic dysfunction and circuit excitability	Human KCNJ10 mutations cause epilepsy-containing EAST/SeSAME syndrome (Bockenhauer et al., 2009). 2-D08 activates Kir4.1 and is neuroprotective/remyelinating in EAE models (Liu et al., 2025b)	Substantial mechanistic rationale but no established antiseizure or antiepileptogenic efficacy; direct MetS→Kir4.1 → PV-PNN evidence is lacking

The candidate hubs listed here are intended as experimentally tractable nodes within the proposed immunometabolic synaptopathy framework rather than as clinically validated therapeutic targets for MetS-associated epilepsy. Representative compounds are included to illustrate current pharmacological tractability and developmental status; their inclusion does not imply established antiseizure, antiepileptogenic, or disease-modifying efficacy in MetS-associated epilepsy. Evidence supporting these targets is derived from heterogeneous experimental and clinical contexts and should not be interpreted as direct validation of the complete MetS-to-epilepsy pathway. KCNJ10 encodes the inwardly rectifying potassium channel Kir4.1.

### 5.2 Implications for experimental intervention

The principal therapeutic value of the present framework is not to define a specific treatment regimen, but to identify intervention points through which the immunometabolic synaptopathy hypothesis can be experimentally tested. The proposed pathway spans multiple biological levels, from systemic metabolic stress and CNS-interface dysfunction to glial reprogramming, extracellular homeostatic failure, and PV-PNN vulnerability. Consequently, interventions at different levels of the pathway can be used to test whether individual components are necessary, reversible, or sufficient to modify downstream circuit susceptibility.

Upstream metabolic manipulation provides one experimental approach. Dietary modification, glycemic control, weight reduction, or pharmacological improvement of systemic metabolism may reduce the magnitude of chronic metabolic and inflammatory exposure. Metabolism-modifying interventions with established relevance to epilepsy, such as ketogenic diets, additionally affect several pathways represented in the model, including NLRP3 activity, mTOR signaling, mitochondrial metabolism, and gut-microbial function (McDaniel et al., 2011; Youm et al., 2015; Olson et al., 2018). However, improvement in seizure control after such interventions should not by itself be interpreted as evidence that the complete immunometabolic synaptopathy pathway has been reversed.

A complementary strategy is pathway-specific intervention at the candidate molecular hubs described in Section 5.1. Pharmacological or genetic modulation of NLRP3, mTOR, HIF-1α, MMP-9, or Kir4.1 could be used to determine whether individual nodes contribute causally to glial sensitization, extracellular homeostatic dysfunction, PV-PNN instability, or seizure susceptibility. In this context, currently available compounds are most valuable as mechanistic tools rather than as established therapies for MetS-associated epilepsy.

Importantly, the present framework does not provide sufficient evidence to recommend specific multidrug combinations. Theoretical complementarity between targets does not establish pharmacological synergy, therapeutic efficacy, or acceptable safety. Combination approaches should therefore be considered only after target dependence and temporal relationships have been demonstrated in appropriate experimental models.

Thus, the immediate therapeutic significance of the framework is primarily experimental: it provides defined intervention points through which the hypothesis can be supported, refined, or falsified.

## 6 Conclusions and future directions

We propose immunometabolic synaptopathy as a hypothesis-driven framework linking chronic metabolic dysfunction to increased epileptogenic susceptibility. In this model, MetS-associated metabolic and inflammatory signals challenge CNS interfaces, promote trained-immunity-like microglial priming and astrocytic homeostatic dysfunction, and may ultimately reduce the resilience of the PV interneuron–perineuronal net (PV-PNN) unit. The novelty of this framework lies not in any single component, but in integrating peripheral metabolic stress, CNS-interface dysfunction, glial reprogramming, extracellular-matrix remodeling, and inhibitory-circuit vulnerability into a unified and testable model.

Importantly, the complete sequence from MetS to persistent glial reprogramming, PV-PNN dysfunction, and spontaneous epilepsy has not been demonstrated end-to-end. The PV-PNN unit should therefore be regarded as a candidate convergence point, and trained-immunity-like microglial priming as a testable mechanistic hypothesis rather than an established disease mechanism. Future studies should determine whether these events occur in a defined temporal sequence, whether persistent microglial changes remain after metabolic normalization, and whether cell- or pathway-specific interventions can prevent downstream circuit dysfunction. Clinically, common obesity, insulin resistance, type 2 diabetes, and MetS should not be conflated with the ILAE category of metabolic epilepsy; in most patients, they are more appropriately considered potential susceptibility modifiers or comorbid metabolic contexts (Scheffer et al., 2017). Ultimately, longitudinal experimental and human studies will be required to determine whether immunometabolic synaptopathy represents a genuine causal pathway, a susceptibility state relevant to selected patients, or primarily a useful conceptual framework for organizing currently fragmented evidence.

## Data Availability

The original contributions presented in the study are included in the article/supplementary material, further inquiries can be directed to the corresponding author.

## Glossary

ADAMTS: a disintegrin and metalloproteinase with thrombospondin motifs
AGEs: advanced glycation end-products
ALK5: activin receptor-like kinase 5
AMPK: AMP-activated protein kinase
AMPA: α-amino-3-hydroxy-5-methyl-4-isoxazolepropionic acid receptor
AQP4: aquaporin-4
BBB: blood–brain barrier
BCG: Bacillus Calmette–Guérin
BDNF: brain-derived neurotrophic factor
CNS: central nervous system
CSF: cerebrospinal fluid
DAMPs: damage-associated molecular patterns
E/I: excitation/inhibition
EAAT1/2: excitatory amino acid transporters 1 and 2
EAE: experimental autoimmune encephalomyelitis
ECM: extracellular matrix
ErbB4: Erb-B2 receptor tyrosine kinase 4
GABA: γ-aminobutyric acid
GDNF: glial cell line-derived neurotrophic factor
GLT-1: glutamate transporter 1
GLUT1: glucose transporter 1
GPR41/43/109A: G protein-coupled receptors 41
43: and 109A
H3K4me3: trimethylation of histone H3 lysine 4
H3K27ac: acetylation of histone H3 lysine 27
HIF-1α: hypoxia-inducible factor 1-alpha
HK2: hexokinase 2
IL: interleukin
KCC2: K^+^/Cl^−^ cotransporter 2
KCNJ10: potassium inwardly rectifying channel subfamily J member 10
Kir4.1: inwardly rectifying potassium channel 4.1
LDHA: lactate dehydrogenase A
LPS: lipopolysaccharide
MetS: metabolic syndrome
MMP-9: matrix metalloproteinase-9
mTOR: mechanistic target of rapamycin
NADPH: nicotinamide adenine dinucleotide phosphate
NF-κB: nuclear factor kappa B
NMDA: N-methyl-D-aspartate receptor
NLRP3: NLR family pyrin domain containing 3
oxLDL: oxidized low-density lipoprotein
PFKFB3: 6-phosphofructo-2-kinase/fructose-2,6-bisphosphatase 3
PNN: perineuronal net
PV: parvalbumin
Qalb: cerebrospinal fluid-to-plasma albumin quotient
RAGE: receptor for advanced glycation end-products
ROS: reactive oxygen species
SCFAs: short-chain fatty acids
SFAs: saturated fatty acids
TCA: tricarboxylic acid cycle
TGF-β: transforming growth factor-beta
TLR4: Toll-like receptor 4
TNF-α: tumor necrosis factor-alpha
TrkB: tropomyosin receptor kinase B
TSC: tuberous sclerosis complex
VEGF-C: vascular endothelial growth factor C
VEGFR3: vascular endothelial growth factor receptor 3
ZO-1: zonula occludens-1.
